# The crowding out of conventional electricity generation by renewable energy sources: implications from Greek, Hungarian, and Romanian electricity markets

**DOI:** 10.1007/s11356-023-30564-y

**Published:** 2023-11-08

**Authors:** Marko Halužan, Miroslav Verbič, Jelena Zorić

**Affiliations:** 1 Holding Slovenske Elektrarne, Koprska ulica 92, 1000 Ljubljana, Slovenia; 2 School of Economics and Business, University of Ljubljana & Institute for Economic Research, Kardeljeva ploščad 17, 1000 Ljubljana, Slovenia; 3 School of Economics and Business, University of Ljubljana, Kardeljeva ploščad 17, 1000 Ljubljana, Slovenia

**Keywords:** Renewable energy sources, Merit order effect, Data mining, Day-ahead electricity prices, Central and South East Europe

## Abstract

To achieve ambitious energy-climate targets, all EU member states have introduced policies to support the market introduction of renewable energy sources (RES) generation. Motivated to close the gap of the merit order effect (MOE) in less mature Central and South East European electricity markets, we empirically confirm economic theory predictions that in the short run, an increase in RES generation reduces electricity prices. The merit order effect is initially econometrically confirmed and quantified. Different econometric model specifications are estimated to differentiate the MOE caused by wind and solar generation and to differentiate the MOE on high-load and low-load days. In addition, we simulate the adjustment of the realised day-ahead electricity prices to the no-RES generation scenario. Modern statistical methods are applied to bridge the gap in the limited public data availability to solve simulation models used in the power system or agent-based simulations. A family of data mining algorithms is applied for the merit order estimation used in the dynamic adaptation of the generation mix to the omitted RES generation. The estimated energy imbalance caused by the excluded RES generation is therefore compensated by the additional conventional generation dispatch according to the estimated power plant merit order. The estimated supply curves for each generation technology assist the reasoning behind the established MOE in econometric models. Based on our findings, policymakers should prioritise policies that facilitate the integration of RES into their electricity markets, which would in turn accelerate energy transition. With increasingly growing shares of renewables in the system, the governments need to rethink the support scheme, where the emphasis should be placed on efficiently integrating renewables in the power system by taking into account temporal and spatial dimensions.

## Introduction

Growth in electricity generation from renewable energy sources (RES) to achieve a less polluting and import-dependent energy sector in the EU member states has influenced electricity market dynamics. National promotion strategies triggered by the Directive 2001/77/EC on renewable energies in the electricity sector have been the major driving force for this development. All EU member states have introduced policies to support the market introduction of RES (Ragwitz and Held 2007). Guaranteed feed-in-tariffs have been most successful to stimulate investments in renewable energies, as investors receive their income on the basis of the set up renewable promotion scheme and not from the electricity sold on spot markets with highly volatile prices (Sensfuß et al. 2008). Consequently, increased renewable generation of electricity crowds out other high(er) marginal-cost technologies and results in lower electricity prices in the wholesale electricity market (Keles et al. 2013). The crowding out of generation from conventional (non-renewable) energy sources with higher marginal costs is recognised in the literature as a merit order effect (MOE). Lower prices result from the fact that renewables bid into wholesale electricity markets at almost-zero prices, and therefore shift the electricity supply curve to the right (Keles et al. 2013). The MOE is in general a heavily researched topic of interest that aims to explain the reduction of marginal costs of energy due to the penetration of renewable sources evidenced from wholesale electricity markets. Past researches have explored the evidence of the MOE for several countries, such as in the context of Germany (Cludius et al. 2014), for Portugal and Spain (Figueiredo and da Silva 2019), for India (Pradhan et al. 2021), for the Czech Republic (Luňáčková et al. 2017), for Italy (Clò et al. 2015), and for Australia (McConnell et al. 2013).

The novelties of our paper are twofold. First, an empirical analysis is conducted in order to confirm and quantify merit order effect in Hungarian, Romanian, and Greek electricity markets. While the phenomenon of renewable energy sources displacing conventional generation has been extensively studied in advanced EU energy markets, we extend the analysis to investigating the MOE in less mature Central and South East European day-ahead electricity markets. This expansion is crucial as it enriches the understanding of MOE in regions with evolving energy transitions. There exist relatively few studies that address the presence of the merit order effect in less mature EU markets, e.g. the merit order effect in the Greek electricity market has been studied by Loumakis et al. (2019), who modelled the electricity demand curve and the electricity production separately in the day-ahead market to analyse the effects of renewable penetration on wholesale electricity prices. Second, by utilising modern statistical methods, the research offers a framework that simulates electricity price adjustments in response to scenarios devoid of RES generation, overcoming data limitations and enhancing the accuracy of simulation models. This methodology innovation has the potential to bridge data gaps and yield insights akin to more complex power system or agent-based simulations, thus further advancing applied power market research.

The analysed electricity markets of Central and South East Europe given their characteristics qualify for a merit order effect analysis. Greek and Romanian electricity markets have higher RES generation shares in their electricity generation mix, and clearly qualify as interesting case study examples. In contrast, Hungary has a low share of renewable generation and serves as a control country. Due to its direct interconnection to the Romanian market, it could be considered as a natural price cap for the expected Romanian prices in the no-RES generation scenario simulation. We expect to confirm that the increase in RES generation crowds out conventional generation sources and in the short run reduces the price of electricity. Further, based on the no-RES generation simulation results, we investigate the effect of RES generation on the electricity price levels, price volatility, and electricity net export.

The empirical MOE analysis is executed to supplement the existing literature focused on key EU energy areas in terms of installed renewable capacity and electricity market development. Prior studies considering empirical confirmation and quantification of the MOE typically address the German (Neubarth et al. 2006; Sensfuß et al. 2008; Weigt 2009; Würzburg et al. 2013; Benhmad and Percebois 2018), Spanish (Sáenz de Miera et al. 2008; Gelabert et al. 2011; Gil et al. 2012; Figueiredo and Silva 2019), and Danish (Jónsson et al. 2010; Unger et al. 2018) electricity markets. Based on the literature review, there is no similar study investigating the MOE in the EU member countries in Central and South East Europe regions.

In this paper, we empirically confirm and quantify MOE by a multivariate regression model similar to Würzburg et al. (2013) analysing the MOE in the German and Austrian electricity markets. Going beyond the existing empirical methodology, we also simulate adjusting the realised day-ahead electricity prices to the no-RES generation scenario. For the preparation of simulated no-RES generation scenario, we have further estimated the influence of RES generation on the country electricity net export and aggregated supply curves for different electricity generation technologies. Part of the domestic RES generation is typically exported to neighbouring countries. Therefore, only a domestically absorbed RES generation share causes MOE and reduces domestic electricity prices. The influence of RES generation on country electricity net export is quantified by a multivariate regression model. According to the economic theory, supply curve quantity and price pairs are determined by the short-run marginal costs of different electricity generation technologies. Aggregated supply curve for the individual generation technology is estimated based on the observed day-ahead electricity prices and reported electricity dispatch. Due to the prominent non-linear behaviour of the electricity price signals (Weron 2014), we estimate aggregated supply curves by employing data mining algorithms. The estimated energy imbalance caused by the excluded renewable generation is compensated by the additional conventional generation dispatch. The required additional conventional generation dispatch to maintain energy balance is priced according to the estimated aggregated supply curves. Based on the no-RES generation simulation results, we can study the effect of RES generation on electricity price levels and volatility.

The paper is structured as follows. “Merit order effect” section looks more closely into the MOE theory and provides literature review. “Methodology” section outlines the methodology and its application. Data, data availability, and country electricity generation mix features are summarised in the “Data” section. “Results and discussion” section reports and discusses the empirical results. Finally, “Conclusion” section presents concluding remarks with summarised key research findings.

## Merit order effect

**Fig. 1:**
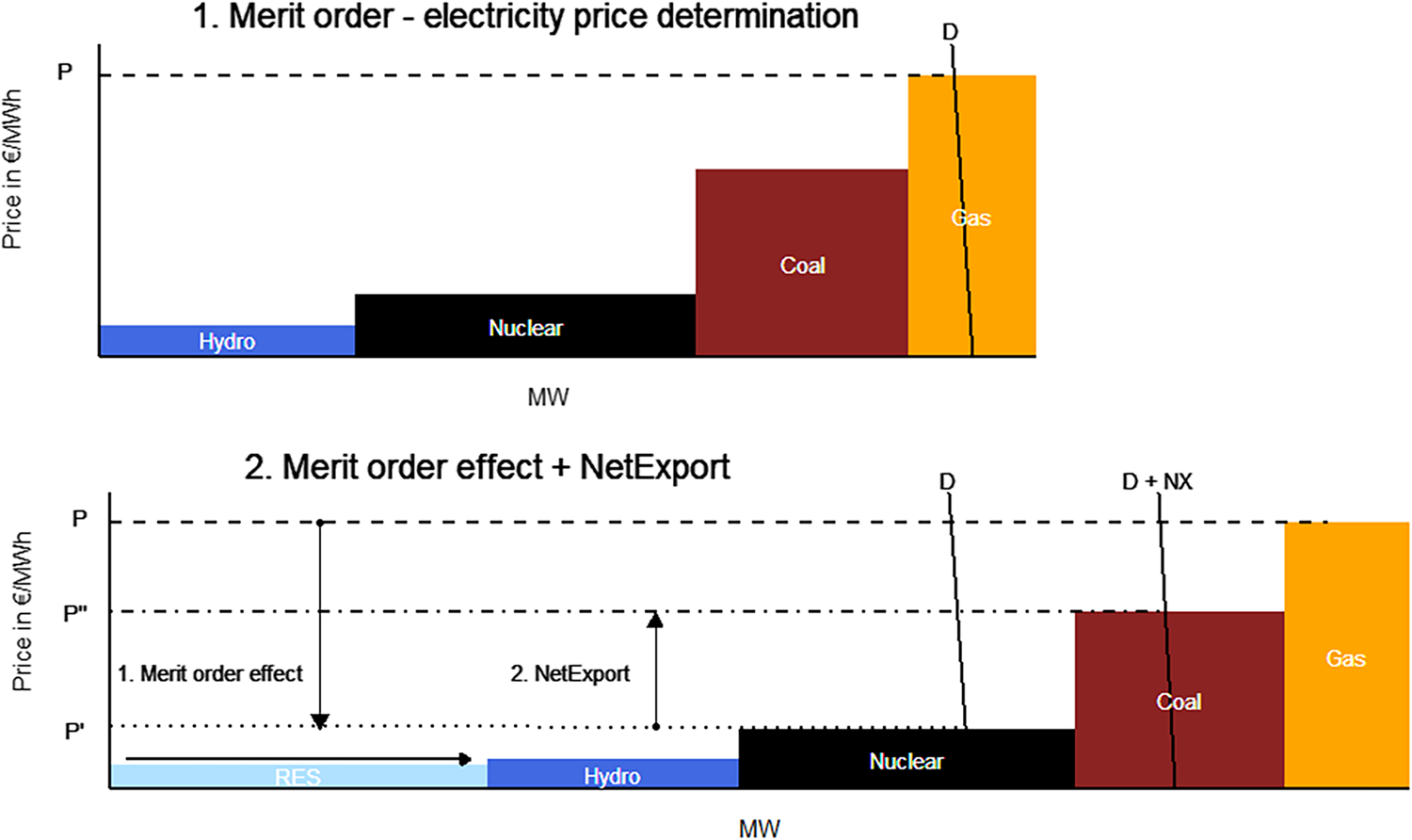
Merit order based on marginal costs, merit order electricity price setting, and merit order effect

Guaranteed feed-in-tariffs support for RES electricity generation has led to growth in the installed capacity of supported technologies. Throughout the article, wind and solar electricity generation are addressed by the RES electricity generation. Theoretical consideration introduced by Jensen and Skytte (2002) suggests that renewable electricity generation results in lower electricity prices. Electricity price is determined at the intersection of the aggregated demand and supply curves. Electricity is an essential commodity, and as such, in the short term exhibits inelastic demand (vertical line *D* in Fig. 1) (Cerjan et al. 2013). The profile of the supply curve is defined by the ranking of the generation units by their short-run marginal costs in increasing order, together with the dispatched energy, in a merit order (Sensfuß et al. 2008). In Fig. 1, electricity price is determined at the price level *P* at the intersection with the gas power plant short-run marginal costs (Fig. 1; 1. Merit order − electricity price determination).

The price reducing impact is called a “merit order effect” and can be explained with the right shift of the supply curve when RES generation with low variable costs is integrated into the supply curve (Fig. 1). Assuming an inelastic demand, electricity price as an intersection between supply and demand will thus decrease to price *P*′ associated with the short-run marginal costs of nuclear technology (Fig. 1; 2. Merit order effect + NetExport). The gradient of the supply curve depends mainly on technologies, efficiencies, fuel prices, start-up costs, and ${\mathrm{CO}}_{2}$ price (Keles et al. 2013).

The shift towards renewable energy can also be attributed to other factors, i.e. ${\mathrm{CO}}_{2}$ prices, policy measures, and human development. The response of electricity demand to price changes is discussed by Fleschutz et al. (2021) for a wide sample of European countries. They show that an increasing carbon price generally elevates the estimated merit order effects. Thus, they conclude that price-based demand response can be an effective economic and environmental improvement tool. Other factors contributing to higher renewable energy consumption were studied for the sample of BRICS countries (Sachan et al. 2023). The authors find that the factors related to environmental policy stringency and human development statistically significantly and positively contribute to the demand for renewable energy.

Electricity interconnections have become increasingly common as a means of integrating electricity markets (Macedo et al. 2021). In general, countries tend to (net) export greater amount of electricity if domestic RES generation increases (Croonenbroeck and Palm 2020). In Fig. 1, this is illustrated with the increase of electricity price from *P*′ to *P*″. Price movement from *P*′ to *P*″ is induced by the foreign demand (*NX*) for cheaper electricity due to the MOE, which increases the final demand for electricity (*D* + *NX*). New electricity price *P*″ is associated by the short-run marginal costs of coal electricity generation technology.

Table 1 summarises day-ahead electricity prices in the analysed period. The influence of German electricity prices on electricity prices across the other regions is confirmed in many studies (Bunn and Gianfreda 2010; Lindström and Regland 2012; Ziel et al. 2015). Table 1 confirms this stylised fact, as the electricity price levels of the analysed perimeter follow German price dynamics.

**Table 1:** Day-ahead electricity prices in €/MWh

Year	DE	HU	RO	GR
2015	31.8	40.6	36.4	51.9
2016	29.0	35.5	33.4	42.8
2017	34.2	50.4	48.2	54.7
2018	44.5	51.0	46.5	60.4

Source: ENTSO-E TP (2020)

**Fig. 2:**
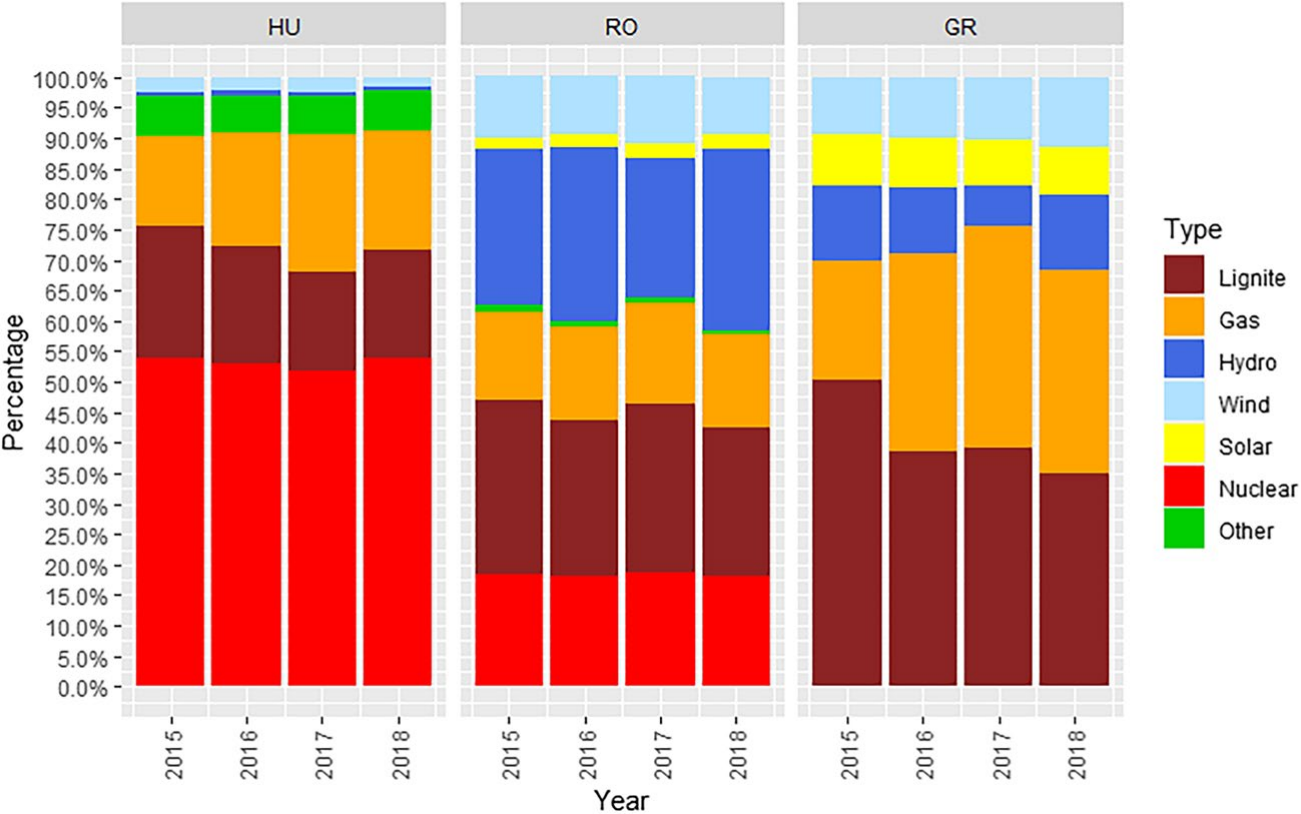
Electricity generation mixes in percentages. Source: ENTSO-E TP (2020)

Figure 2 presents electricity generation mixes of Hungarian, Romanian, and Greek power systems. RES have insignificant contribution to the Hungarian generation mix, as there is no solar generation, whereas wind accounts for less than 2.5% of total generation on a yearly basis. In the Romanian generation mix, on average, 10% of electricity is generated by the wind and 2.5% in solar power plants. In Greece, 10% and 7.5% of electricity are generated by the wind and solar power plants, respectively. Based on the observed wind and solar maximum generation outputs in the analysed power systems, we can conclude that the installed RES capacity remained relatively constant over the 2015–2018 period (Table 4 of the Appendix). Romania with 12.5% and Greece with 17.5% RES share in generation mix clearly qualify as good candidates for the MOE analysis.

According to Würzburg et al. (2013), key MOE studies can be classified in simulation-based and empirical analysis studies. Simulation studies are based on the simulation models (e.g. unit commitment model) using real past or hypothetical data, whereas empirical studies are generally performed with econometrics models on real past data. Due to fundamental difference in approaches, drawing general conclusions and result comparison of different papers should be done with special care.

Important simulation-based studies typically rely on information rich and flexible simulation models used for power system or agent-based simulations. With an agent-based simulation platform, Sensfuß et al. (2008) analysed German electricity prices with and without RES generation. RES generation caused a price reduction by 1.7 to 7.8 €/MWh. Woll and Weber (2012) simulated the German electricity system by 34 generation technologies for electricity generation, fuel prices, and ${\mathrm{CO}}_{2}$ prices. In the no-RES generation, scenario electricity prices are 4.04 €/MWh higher compared to the base scenario with wind generation. Fürsch et al. (2012) simulated merit order effect for Germany based on the DIME model (Dispatch and Investment Model for Electricity Markets in Europe). This model accounts for the international flows and dynamic adaptation of generation mix to increased RES generation. Due to the predicted RES generation growth, they predicted in years 2015, 2020, 2025, and 2030 a price reduction of 2 €/MW, 4 €/MWh, 5 €/MWh, and 10 €/MWh, respectively. Sáenz de Miera et al. (2008) report in the Spanish market simulation analysis between years 2005 and 2007 a price reduction caused by the wind generation of 7.08 €/MWh to 12.44 €/MWh.

With the increased market transparency and ex-post data availability, number of published empirical studies quantifying the impact of RES generation on electricity prices significantly increased. Neubarth et al. (2006) estimated by the univariate econometric model the impact of wind generation on German electricity day-ahead prices in years 2004 and 2005. They find that the electricity price drops by 1.89 € for each additional GW of wind power generation. Using time-series regression analysis, Cludius et al. (2014) estimated a price drop caused by RES generation in Germany by 6 €/MWh in 2010, 10 €/MWh in 2012, and a projected price drop of 14–16 € in year 2016. Furthermore, Deane et al. (2017) conducted an ex-post and ex-ante study of the influence of renewable energy on energy prices in European countries over the past few years and between 2030 and 2050. They estimated that the electricity prices in Europe would generally decline over the medium term as renewable energy production will likely increase the supply of electricity.

Macedo et al. (2021) estimated by using a SARMAX/GARCH time-series econometric approach the impact of RES generation and net export on Swedish day-ahead electricity prices from 2016 to 2020. They estimated a model for each hour of the day individually and confirmed homogenous negative impact of RES generation on electricity price. A 1% increase in RES generation decreased the electricity price by 0.0609%. Macedo et al. (2020) expanded preceding study to the Portugal electricity market. They estimated that the 1% increase in RES generation on average decreased the Portuguese electricity price by 0.056%. Figueiredo and Silva (2019) based on historical Spain and Portuguese (Iberian market) electricity power exchange data quantified the MOE with the GARCH econometric model. For the period from 2013 to 2017, they confirmed a MOE of 13.11 €/MWh for wind generation and 8.79 €/MWh for solar generation.

Azofra et al. (2014) estimated the MOE in the Spanish electricity market using a data mining regression tree (M5P) algorithm. For year 2012, they have estimated a price drop between 7.42 and 10.94 €/MWh caused by the wind generation. Clò et al. (2015) estimated the MOE of wind and solar generation in the Italian power market. They have reported that in the years 2005–2013 for each additional GW of solar and wind generation the electricity prices on average dropped by 2.3 €/MWh and 4.2 €/MWh, respectively. Janda (2018) investigated the influence of solar generation on Slovak day-ahead electricity price in years 2011–2016. The estimated multivariate model indicates that, ceteris paribus, 1% increase in solar generation is associated with a spot price decrease from 0.016 to 0.067%. Given the literature review of the most important studies on MOE in European electricity markets, we aspire to close the gap of yet unresearched MOE in Central and South East European electricity markets.

## Methodology

The presence of MOE in Hungary, Greece, and Romania is initially statistically verified by a multivariate regression model. Then, we simulate the adjustment of the realised electricity prices to the no-RES generation scenario. The applied no-RES generation simulation approach intuitively takes as an example the DIME model (Dispatch and Investment Model for Electricity Markets in Europe) used by Fürsch et al. (2012). The DIME model accounts for the international flows and dynamic adaptation of the generation mix to changes in RES generation.

### Econometric merit order effect verification

To statistically verify the presence of the MOE, we estimate a multivariate regression model similar to Würzburg et al. (2013). Neubarth et al. (2006) found that with daily average values RES explanatory variables tend to be more relevant for the definition of day-ahead prices in the German market area. Therefore, to eliminate ad hoc anomalies and short-term noise, all model variables are calculated as the daily average values. In Eq. 1, electricity price ($${P}_{elec,d})$$ is the dependent variable, whereas the explanatory variables are the previous day electricity price ($${P}_{elec, d-1})$$, realised German electricity price ($${P}_{DE,d}$$), the demand for electricity ($${Load}_{d}$$), wind and solar generation ($${RES}_{d}$$), the net export of electricity ($${NX}_{d}$$), and standard error term ($${\varepsilon }_{d})$$. In Eq. 1, Δ represents the first difference operator and $$d$$ stands for daily observations:

1 $$\Delta {P}_{elec,d}={\beta }_{o}+{{\beta }_{1}\Delta P}_{elec, d-1}+{\beta }_{2}\Delta {P}_{DE,d}+{\beta }_{3}\Delta {Load}_{d}+{{\beta }_{4}\Delta RES}_{d}+{\varepsilon }_{d}$$

According to Weron (2014), AR-type models provide the backbone of all time-series electricity price models; therefore, the autoregressive explanatory variable ($${P}_{elec, d-1})$$ is used in the model. The German–Austrian market features an important renewable capacity that is obviously related to the strong renewable support scheme that has been in place for many years (Würzburg et al. 2013). Explanatory variable $${P}_{DE, d}$$ is added into the models, as the influence of German electricity price on electricity prices across other regions is confirmed in other studies (Bunn and Gianfreda 2010; Lindström and Regland 2012; Ziel et al. 2015). Germany is a highly developed economy where the energy markets are linked either through substitution possibilities for consumers or through input factor influences (such as gas-fired power plants) (Würzburg et al. 2013). For the studied perimeter, we could not find appropriate public coal and gas price indexes; therefore, variable $${P}_{DE, d}$$ is used as an indicator for the fuel and CO2 price levels in Greek, Hungarian, and Romanian markets. The electricity demand $${Load}_{d}$$ is inelastic, but with high seasonality and sensitivity to weekly patterns of consumption. The MOE in Eq. (1) is controlled by the variable $${RES}_{ d}$$ measuring the daily wind and solar electricity generation.

### Electricity price simulation in the no-RES generation scenario

In the no-RES generation simulation, we adjust observed hourly electricity prices by eliminating present merit order effect and adjusting net export levels. Therefore, simulation requires estimation of the power plant merit order and electricity export dependency on RES generation. In the no-RES generation scenario, an additional quantity that must be supplied from the conventional power plants is equal to the sum of realised RES generation and net export implied by the RES generation (foreign demand for cheaper energy). The power system characteristic is that the electricity supply and demand must always be balanced. Therefore, we can equate the required volume of additional conventional generation to secure the power system balance in the simulated no-RES generation scenario by Eq. 2:

2 $${\Delta ConventionalSupply}_{h}={\Delta Load}_{h}+{\Delta NX}_{h}-{\Delta RES}_{h}$$

The electricity demand is deemed to be inelastic; therefore, we can reconstruct the realised intersection of the aggregated supply and demand curve as a function of the observed electricity day-ahead price and the estimated merit order of the system power plants. The simulated electricity price in the no-RES scenario corresponds to a shift of the supply and demand curve. The left shift of the supply curve is equal to the realised RES generation, whereas the demand shift is equal to the estimated change in the net export ($\Delta \widehat{NX}$) triggered by the RES generation. Therefore, estimated energy imbalance ($\widehat{EnergyImbalance}$) caused by the excluded RES generation is filled by the additional conventional generation supply, according to Eq. 3:

3 $${\widehat{EnergyImbalance}}_{h}=\Delta {\widehat{NX}}_{h}-{\Delta RES}_{h}$$

The estimated ${\widehat{EnergyImbalance}}_{h}$ is the required additional conventional supply that secures the power system balance and is priced according to the estimated system merit order. The simulated market clearing electricity price is equal to a price in the last price-quantity pair that fills estimated $\widehat{EnergyImbalance}$ quantity.

#### Impact of the RES generation on electricity net export

Traber and Kemfert (2009) confirmed that the neighbouring countries with lower RES generation in their generation mix (high ${\mathrm{CO}}_{2}$ intensity) benefit by the electricity imports from countries with higher RES generation in the generation mix (low ${\mathrm{CO}}_{2}$ intensity). The impact of RES generation on net export is estimated by the multivariate regression model (Eq. 4). According to economic theory, the electricity net export should be lower in no-RES generation scenarios. Therefore, it is crucial to quantify the impact of RES generation on electricity net export and account for it in the no-RES generation scenario. In Eq. 4, electricity net export (${NX}_{h}$) is the dependent variable, whereas the explanatory variables include the 24-h-lagged electricity net export $({NX}_{h-24})$ and the realised wind and solar generation (${RES}_{h}$), whereas ${\varepsilon }_{h}$ represents the standard disturbance term. In the multivariate regression model, defined by Eq. 4, Δ represents the first difference operator and $h$ stands for the hourly observations:

4 $$\Delta {\widehat{NX}}_{h}={\beta }_{o}+{{\beta }_{1}\Delta NX}_{h-24}+{\beta }_{2}\Delta {RES}_{h}+{\varepsilon }_{h}$$

The influence of RES generation on net export, i.e. international trade, is controlled by the ${NX}_{h}$ variable calculated as a sum of all country inflows and outflows (Eq. 5):

5 $${NX}_h=\sum\nolimits_i^I({Inflow}_i-{Outflow}_i)\;\mathrm{where}\;i\in\left\{{Border}_1,\dots,{Border}_I\right\}$$

The impact of RES generation on net export is estimated by the 7-day rolling-window approach over the available data set. Therefore, each model is estimated on 168 hourly data points.

#### Merit order estimation

The ranking of the generation units by their short-run marginal costs in the increasing order, together with the dispatched energy, can be efficiently simulated by the unit commitment models^1^ that minimises the total dispatch costs of the power plant fleet (Schill et al. 2017). For the considered time-period and analysed country scope, we could not obtain the required data.^2^ With our publicly available data source, we were limited to the reported aggregated hourly output for each type of power plants (presented in Fig. 2) and hourly day-ahead power prices. Therefore, we model short-term economic dispatch of gas, lignite, nuclear, and other power plants on the aggregate level. Due to the low marginal costs of generation, the supply of the hydro and nuclear technology is predominantly defined by the hydrology levels and nuclear availability. The economic dispatch of the hydro and nuclear power plants in the no-RES generation scenario is due to low marginal costs of generation deemed to be unchanged. The merit order of gas, lignite, nuclear, and other power is modelled according to Eq. 6, where ${P}_{elec,i,h}$ is the day-ahead electricity price, ${OutputShare}_{i, h}$ is the percentage output of the observed aggregate power plant capacity and ${\varepsilon }_{i, h}$ is the error term. According to the electricity market, economics observed day-ahead electricity price (${P}_{elec,i,h}$) corresponds to the generation marginal costs of the most expensive power plant serving electricity to the market. In Eq. 6, $h$ stands for the hourly observations:

6 $${OutputShare}_{i, h}={\beta }_{o}+{{\beta }_{o}P}_{elec,i,h}+{\varepsilon }_{i, h}\;\mathrm{where}\;i\in \left\{Gas,\;Lignite,\;Other\right\}$$

Non-linear electricity price behaviour fundamentally results from the profile of the supply curve. Therefore, we estimate model defined by Eq. 6 with predictive modelling approaches that can handle such non-linearities. We have estimated the merit order for the distinct types of power plants by three data mining algorithms: the *k*-nearest neighbours algorithm (KNN),^3^ regression tree algorithm (M5P),^4^ and the random forest algorithm (RFR).^5^ Merit order is estimated by the 7-day rolling-window approach over the entire data set. The selected window size is large enough for the unbiased estimation and narrow enough to recognise for the temporary supply features. By the term “temporary supply features”, we specifically address non-accounted variables such as generation availability, fuel prices, ${\mathrm{CO}}_{2}$ prices, start-up costs, and strategic behaviour.

## Data

Data availability and accessibility historically limited applied power market research (Hirth et al. 2018). The situation in Europe has changed in 2015 with the commencement of the Transparency Platform (TP) (ENTSO-E TP 2020) operated by the European Network of Transmission System Operators for Electricity (ENTSO-E). The Hungarian, Greek, and Romanian working data sets span from 1.1.2015 to 30.9.2018, resulting in a time series of 1368 days or 32,832 hourly observations.

With the available ENTSO-E TP data, we were limited to the reported aggregated hourly output for each type of power plants, scheduled commercial exchanges (net export), and hourly day-ahead power prices. In the data collection phase, we noticed that there are missing data points and non-reported data types in the ENTSO-E TP data base. Therefore, the Romanian data set is a blend of ENTSO-E TP data and Romanian national transmission system operator’s data source (Transelectrica 2020) for the reported aggregated actual generation. With the blended data set, we can econometrically confirm the MOE and quantify the RES generation effect on the country’s net exports. The no-RES generation simulation is structured upon the estimated merit order, as we could not find required data for solving unit commitment problem (historical power plant output, fuel prices, start-up costs, efficiency, etc.). Merit order estimation is performed with the family of data mining algorithms that can handle non-linearities associated with the profile of the supply curve and electricity prices. Due to the limited public data availability, the analysed countries can still be classified as less mature power markets.

## Results and discussion

### Econometric merit order effect verification and quantification

Multivariate regression models are estimated to econometrically confirm and quantify merit order effect in Hungary, Greece, and Romania in the period from 2015 to 2018-Q3. For each country, we have estimated eight model specifications to quantify and confirm MOE. Model specifications 1–4 are estimated on the individual calendar year data samples. Estimation on such data samples is done to observe possible differences due to varying penetrations of renewable sources and due to possible long-run adjustment of the electricity sector to merit order effects. In Fig. 2, we can observe that the electricity generation mix shares are varying in the analysed period. In all countries, we can observe a tendency towards less lignite generation share in the generation mixes. With such an analysis setting, we can detect the influence of generation shares in generation mixes on the price effects of renewable generation over the time.

Model specification 5 is estimated on the whole data sample from year 2015 to 2018-Q3. Table 2 summarises estimation results of model 5 and confirms MOE, i.e. negative impact of increased RES generation (*∆Ren)* on electricity prices. Model specification 6 is estimated to differentiate the impact of solar and wind generation on the observed day-ahead electricity prices. This is done by the use of separate coefficients that intend to identify the different generation patterns of these technologies (Würzburg et al. 2013). Econometrically estimated quantitative MOE is interpreted as a price reduction in €/MWh for each additional GWh of renewable generation. Würzburg et al. (2013) reported that much higher price effects are reported for smaller power systems compared to larger power systems, as the 1 GWh of additional electricity generation presents much higher generation share in smaller systems. Models 7 and 8 are estimated on data samples of upper quarter of high-load days and the lower quarter of low-load days. This is done to verify economic theory that due to the steep profile of merit order curve when the electricity system is close to full capacity, RES generation has much higher impact on the electricity price reduction. This phenomenon is observed and confirmed in Gelabert et al. (2011), Jónsson et al. (2010), and Würzburg et al. (2013). Estimation results for different model specifications for the Greek electricity market are reported in Table 5, for the Hungarian electricity market in Table 6, and for the Romanian electricity market in Table 7 of the Appendix.

**Table 2:** OLS estimation of daily changes in electricity prices (2015–2018-Q3)

Model 5	GR	HU	RO
	*∆Pelec, t*	*∆Pelec, t*	*∆Pelec, t*
*∆Pelec, t* − 1	0.704 [0.00***]	0.582 [0.00***]	0.549 [0.00***]
*∆DE, t*	0.160 [0.00***]	0.313 [0.00***]	0.283 [0.00***]
*∆Load, t*	0.001 [0.00***]	0.006 [0.00***]	0.004 [0.00***]
*∆Ren, t*	− 0.004 [0.00***]	− 0.013 [0.00***]	− 0.007 [0.00***]
*R*^2^	0.77	0.75	0.72
Adjusted *R*^2^	0.77	0.75	0.72
*F*-test	1127.29	994.96	846.03
*p* value (*F*)	0.00	0.00	0.00

^***^ indicating significance at 1% level, and *p* values in [] brackets

Model specification 5, estimated on the whole data sample, confirms the MOE presence in all three countries. The coefficients of renewable generation reported in Table 2 are negative and statistically significant. Greek day-ahead electricity price decreases ceteris paribus by 4 €/MWh for each additional GWh produced by the RES. Ceteris paribus, the Hungarian day-ahead electricity price would decrease by roughly 13 €/MWh, whereas the Romanian electricity price would decrease on average by 7 €/MWh for each additional GWh produced by the RES.

In order to test the econometric validity of the estimates presented in Table 2, we performed some relevant statistical tests; namely, we tested the presence of heteroscedasticity, normality of residuals, multicollinearity of explanatory variables, and residual autocorrelation (results are presented in Tables 8, 9, 10, and 11 in the Appendix). While explanatory variables do not seem to be correlated, we detected the problem of heteroscedasticity and non-normality of residuals. In order to omit the potential effects of heteroscedasticity and non-normality on the significance of results, we estimated the model with the Newey-West robust standard errors. We confirmed the statistical significance of the results (results are presented in Table 12 in the Appendix). Furthermore, we could not reject the presence of residual autocorrelation in the case of Hungary and Romania. Thus, we checked the robustness of the results by including an additional (second) lag of electricity prices in corresponding countries (results are presented in Table 13 in the Appendix). Results also remain robust to changes in the lag structure of estimated regressions.

Model specifications 1–4, estimated for each individual year (2015–2018), confirm the general findings of the model 5. In the Greek electricity market, coefficients associated with the RES generation are always negative and indicate an average decrease of electricity price from 5 to 7 €/MWh for each additional GWh produced by the RES. In Fig. 2, we can observe the highest share of gas generation in the year 2017. The gas price is only significant for the high-load days because of the additional requirements for fossil fuels so that peak-load plants can cope with the unusually high demand (Würzburg et al. 2013). Therefore, the electricity price was frequently set by the expensive gas generation technology. This coincides with the highest econometrically estimated MOE in 2017.

The coefficients associated with RES generation in Hungary are always negative and indicate an average decrease of the electricity price from 5 to 35 €/MWh for each additional GWh produced by the RES. In 2017, the high Hungarian electricity price coincided with the high share of gas generation in that year (Fig. 2). The highest MOE is—similar to the Greek market—estimated by the model specification 3 for the calendar year 2017. The coefficients are statistically significant, except in the model estimated on data for 2018.

Generation shares in the Romanian generation mix are stable (Fig. 2). RES generation coefficients for Romania are always negative and indicate an average decrease of electricity price from 6 to 11 €/MWh for each additional GWh produced by the RES. The highest MOE is estimated by the model specification 1 for the calendar year 2015. Therefore, the highest estimated MOE in the first year could be associated with the lagging adjustment of the electricity sector to merit order effects.

Model specification 6 differentiates the MOE of wind and solar generation. According to Table 4 of the Appendix, the observed maximum solar penetration and wind penetration in Greece are 1.7 GW and 2.1 GW, respectively (ENTSO-E TP). The wind and solar generation coefficients in Greece are negative, similar in levels, and statistically significant. Ceteris paribus, additional GWh of wind generation decreases day-ahead electricity prices approximately by 4 €/MWh, whereas an additional GWh of solar generation reduces electricity prices by 3 €/MWh. As there was no solar generation in Hungary in the analysed period, model specification 6 estimated to differentiate the MOE of wind and solar generation is equivalent to model specification 5. According to Table 4 of the Appendix, the observed maximum solar penetration and wind penetration in Romania are 2.8 GW and 0.9 GW, respectively (ENTSO-E TP). The wind generation coefficient in Romania is statistically significant and negative, while solar generation coefficient is statistically significant and positive. Positive solar generation coefficient indicates higher electricity prices with solar penetration in the generation mix. This is not in line with the economic reasoning outlined in the “Merit order effect” section. Solar generation peaks in summer during day hours, where the electricity prices are due to naturally lower hydro generation availability and higher electricity consumption (air conditioning) typically higher. This positive correlation between the summer solar generation and electricity prices might have influenced model estimation. The inclusion of a dummy variable indicating a summer period did not improve the results.

For Greece, the comparison of the MOE on high- and low-load days confirms the findings of previous studies, wherein the MOE is more pronounced for high-load days. The difference between the high-load days (model specification 7) and low-load days (model specification 8) is approximately 2 €/MWh. The estimated coefficients are statistically significant in both model specifications. Similarly, the comparison of MOE on high- and low-load days in Hungary reveals an approximately 5 €/MWh difference in electricity price reduction. For Romania, the obtained results are not completely in line with previous studies, as the estimates indicate higher MOE on low-load days, where the difference between the high- and low-load days is approximately 1 €/MWh.

### Electricity price simulation in the no-RES generation scenario

The adjustment of the realised day-ahead prices to the no-RES generation scenario requires several pieces of analysis. Firstly, it is crucial to quantify the impact of RES generation on the electricity net export. In the no-RES generation scenario, the electricity net export must be adjusted for the electricity net export share associated with the RES generation. Secondly, merit order estimation is required for the determination of the aggregated demand and supply curve intersection (given the inelastic domestic demand assumption and observed day-ahead electricity price). Based on the estimated energy imbalance caused by the excluded RES generation (Eq. 3), new no-RES generation electricity day-ahead price is determined with a left shift of the estimated merit order and demand shift that is equal to the estimated change in the net export.

Foreign demand for cheaper energy, i.e. the impact of RES generation on net export, is estimated by the multivariate regression model (Eq. 4). The model is estimated by the 7-day rolling-window approach over the available data set. Yearly aggregation of the estimation results for the Hungarian electricity market is reported in Table 14 in the Appendix. The explanatory variable ${RES}_{t}$ illustrates the impact of RES generation on net export. The coefficient has a positive value between 0.43 and 0.85 and is statistically significant. Therefore, 43–85% of the Hungarian net export is RES generation dependent. This is in line with the research findings of Traber and Kemfert (2009) that the neighbouring countries with lower RES generation benefit by the electricity imports from countries with higher RES generation in the generation mix. The estimation summary for the Greek electricity market results in statistically significant coefficients indicating that 4.5–5.5% of the Greek net export is RES generation dependent (Table 15). Further, 46–53% of the Romanian net export is attributed to the RES generation (Table 16). The estimated explanatory coefficients are statistically positive and in line with previous research findings. A more detailed analysis of the RES generation impact on electricity net export is beyond the scope of this research.

**Fig. 3:**
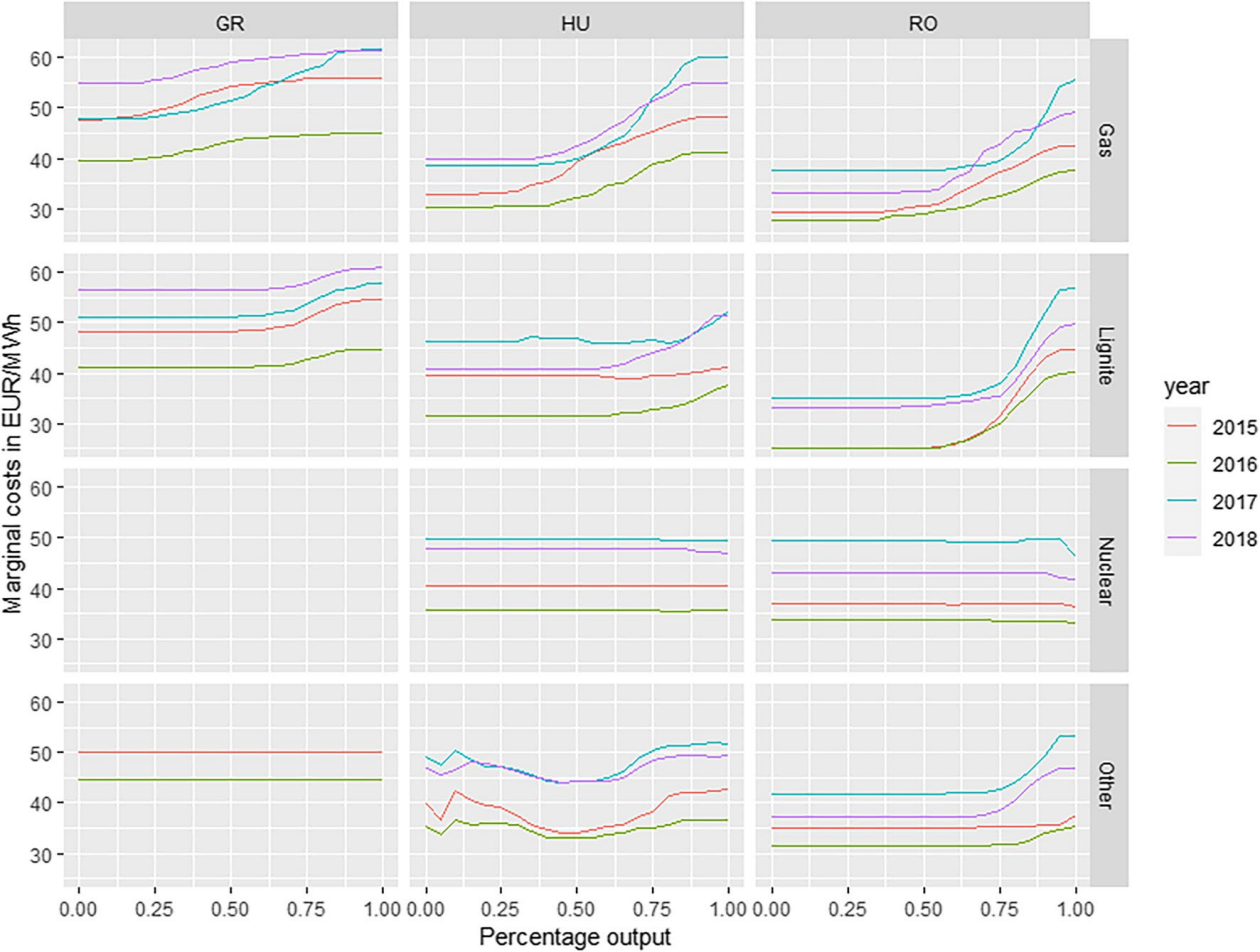
Estimated merit order by the M5P algorithms

**Fig. 4:**
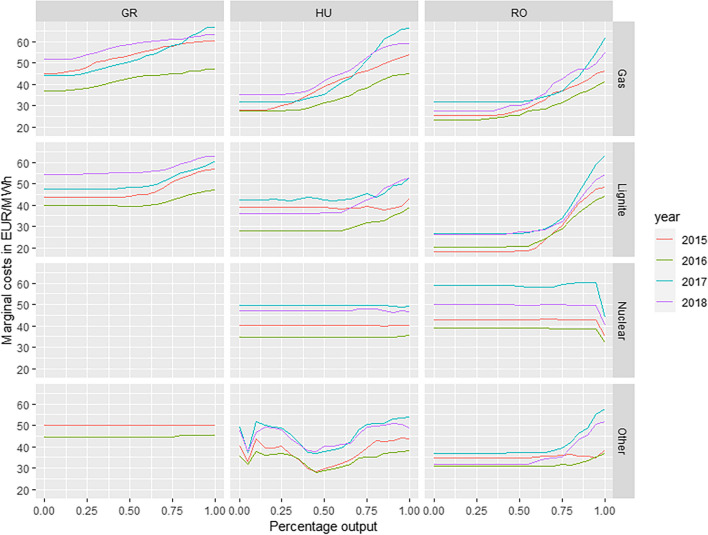
Estimated merit order by the *k*-nearest neighbour algorithm (yearly aggregation of weekly estimates)

**Fig. 5:**
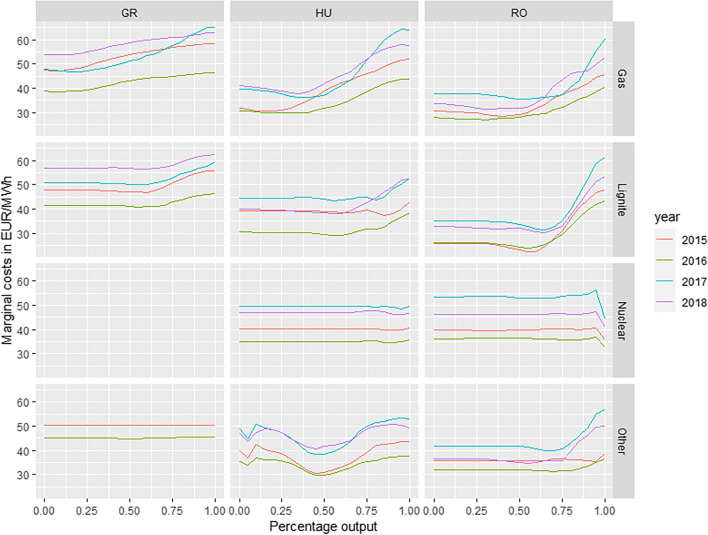
Estimated merit order by the random forest algorithm (yearly aggregation of weekly estimates)

Merit order, i.e. ranking of the generation units by their short-run marginal costs in increasing order, together with the dispatched energy, is estimated by the family of data mining algorithms. Different merit order estimation approaches are applied to assure and confirm simulation robustness. Merit order is estimated by the 7-day rolling-window approach over the entire data set. Figure 3 represents the aggregation of weekly merit order estimations by the regression tree algorithm (M5P). Estimated merit orders by the *k*-nearest neighbours (Fig. 4) and random forest regression algorithms (Fig. 5) have similar shapes compared to the M5P algorithm and are presented in the Appendix. Both algorithms serve as a robustness check and lead to similar results compared to the M5P algorithm. Supply curve shapes are defined by the technology short-run marginal costs. Estimated aggregated short-term economic dispatch of the marginal cost intensive technologies is in accord with the economic reasoning discussed in Schröder et al. (2013).

Estimated supply curves of gas and lignite power plants have the steepest slope, which is expected due to the high fuel and CO_2_ costs.^6^ Conversely, the nuclear supply curve is very stable due to the low marginal costs and limited generation flexibility. The estimated supply curve of the nuclear technology is perfectly elastic at the approximate yearly average electricity price level. This confirms that the nuclear generation variation is especially low and estimating characteristic supply curve is unreasonable. Therefore, nuclear power plant generation is excluded from the merit order used in the no-RES generation scenario.

Power plants classified under the category “other” represent only a small generation share in the analysed scope (Fig. 2). The estimated U-shaped Hungarian curve for other supply is a result of aggregating all other ENTSO-E TP generation types into this category (biomass, fossil oil, other, etc.). The estimated other supply curve for the Greek market is perfectly price elastic (fossil oil; ENTSO-E TP category), whereas for the Romanian market (biomass; ENTSO-E TP category) it is price dependent. The merit order estimated by the *k*-nearest neighbours algorithm is graphically represented in Fig. 4 of the Appendix.^7^ Fig. 5 of the Appendix is a graphical representation of the merit order estimated by the random forest algorithm.^8^

Merit orders estimated by all three algorithms are similar in shape and confirm discussed technology characteristics. The applied modelling approaches are suitable to cope with the non-linear electricity supply curve behaviour. Nuclear power plant generation is confirmed to be stable and does not vary. Due to the low marginal costs of generation, the supply of the hydro and nuclear technology is predominantly defined by the hydrology levels and nuclear availability. The slope of the merit order is defined by the technologies with significant short-term marginal costs of generation. Therefore, the adjusted electricity price in the no-RES generation simulation is determined by the estimated merit order of lignite, gas, and other technology.

Table 3 summarises the results of the no-RES scenario simulation based on the previously analysed impact of RES generation on net export and estimated merit order. The Hungarian realised day-ahead price adjusted to the no-RES generation scenario on average changed insignificantly (Table 3). In year 2015, simulated electricity prices would rise between 0.05 and 0.21 €/MWh, whereas the standard deviation is reduced. Analogous results are established for the year 2016. The reduced standard deviation in the no-RES generation scenario, registered in all simulation years, is a result of eliminated volatile RES generation. Similar to Dong et al. (2019), we confirm that RES generation amplifies electricity price volatility. In contrast, according to the simulation results in years 2017 and 2018, the effect of RES generation on the electricity prices is insignificant. Due to lower RES generation share in the Hungarian generation mix (Fig. 2) and high electricity exports associated with the RES generation (Table 14 of the Appendix), simulation results correspond to the MOE reasoning. Simulation robustness is proved, as the general conclusions do not depend on the selected merit order forecasting algorithm.

**Table 3:** No-RES generation simulation results

Year	Country	Model	Realised prices		Simulated prices		Difference	
			Mean	S.D.	Mean	S.D.	Mean	S.D.
2015	HU	KNN	40.61	236.59	40.77	232.01	0.17	− 4.58
2015	HU	M5P	40.61	236.59	40.66	234.85	0.05	− 1.75
2015	HU	RFR	40.61	236.59	40.82	231.84	0.21	− 4.75
2016	HU	KNN	35.49	171.09	35.52	170.50	0.03	− 0.60
2016	HU	M5P	35.49	171.09	35.49	170.88	0.01	− 0.22
2016	HU	RFR	35.49	171.09	35.54	170.19	0.05	− 0.90
2017	HU	KNN	50.36	580.99	50.36	580.99	0.00	0.00
2017	HU	M5P	50.36	580.99	50.36	580.99	0.00	0.00
2017	HU	RFR	50.36	580.99	50.36	580.99	0.00	0.00
2018-Q2	HU	KNN	46.46	291.18	46.46	291.12	0.00	− 0.07
2018-Q2	HU	M5P	46.46	291.18	46.46	291.13	0.00	− 0.06
2018-Q2	HU	RFR	46.46	291.18	46.46	291.04	0.00	− 0.14
2015	GR	KNN	51.93	121.42	52.75	105.21	0.82	− 16.21
2015	GR	M5P	51.93	121.42	52.13	119.75	0.20	− 1.67
2015	GR	RFR	51.93	121.42	52.89	104.76	0.96	− 16.66
2016	GR	KNN	42.85	81.07	44.13	83.76	1.28	2.69
2016	GR	M5P	42.85	81.07	43.18	80.06	0.33	− 1.00
2016	GR	RFR	42.85	81.07	44.43	82.89	1.58	1.83
2017	GR	KNN	54.68	292.08	57.56	322.83	2.88	30.75
2017	GR	M5P	54.68	292.08	55.53	297.71	0.85	5.63
2017	GR	RFR	54.68	292.08	57.94	332.23	3.26	40.15
2018-Q2	GR	KNN	56.94	103.94	58.96	76.84	2.02	− 27.10
2018-Q2	GR	M5P	56.94	103.94	57.55	94.59	0.61	− 9.36
2018-Q2	GR	RFR	56.94	103.94	59.35	72.48	2.41	− 31.46
2015	RO	KNN	36.43	204.84	37.66	197.59	1.23	− 7.25
2015	RO	M5P	36.43	204.84	36.85	204.64	0.42	− 0.20
2015	RO	RFR	36.43	204.84	38.03	195.57	1.60	− 9.27
2016	RO	KNN	33.37	163.77	34.63	160.33	1.25	− 3.44
2016	RO	M5P	33.37	163.77	33.76	164.20	0.39	0.43
2016	RO	RFR	33.37	163.77	35.01	156.80	1.63	− 6.97
2017	RO	KNN	48.19	575.54	50.34	564.07	2.15	− 11.47
2017	RO	M5P	48.19	575.54	48.88	572.86	0.69	− 2.69
2017	RO	RFR	48.19	575.54	50.91	563.52	2.72	− 12.02
2018-Q2	RO	KNN	41.19	356.62	42.91	343.25	1.72	− 13.37
2018-Q2	RO	M5P	41.19	356.62	41.63	353.76	0.44	− 2.86
2018-Q2	RO	RFR	41.19	356.62	43.26	346.44	2.07	− 10.18

Source: ENTSO-E TP (2020) and own calculations

The Greek realised day-ahead price adjusted to the no-RES generation scenario on average changed between 0.2 and 3.26 €/MWh. Estimated energy imbalance covered by the conventional generation technologies had the highest impact on the electricity prices in simulation years 2017 and 2018. However, in the analysed period, the Greek RES generation share remained steady. Therefore, simulated electricity price peaks in years 2017 and 2018 coincide with the changed estimated merit order profile of gas generation. In years 2017 and 2018, the estimated merit order profiles become much more explicit and with notable slope changes near the full capacity utilisation (Fig. 3). After year 2016, German electricity prices significantly increased. Therefore, changed estimated merit order profile slope is most likely associated with the structural change in the short-term marginal costs structure. The highest electricity price increase between 0.85 and 3.26 €/MWh is simulated in year 2017. Price volatility is on average reduced, though in 2017 simulation results indicate increased price volatility. The lowest hydro generation is reported in year 2017 (Fig. 2), whereas RES generation share remained steady. As a result, market clearing price occurred more frequently at the steepest profile of the supply curve. Low hydrology was compensated by the increased lignite and gas generation (Fig. 2). Therefore, realised RES generation had to some extent stabilising effect on the electricity prices as the simulated standard deviation in no-RES scenario increased. Due to higher RES generation share in the Greek generation mix (Fig. 2) and low electricity exports associated with the RES generation, simulation results correspond to the MOE reasoning.

The Romanian realised day-ahead price adjusted to the no-RES generation scenario on average amounted between 0.39 and 2.72 €/MWh (Table 11 of the Appendix). The estimated energy imbalance covered by the conventional generation technologies had the highest impact on the electricity prices in simulation years 2017 and 2018. In the analysed period, the Romanian RES generation share remained steady. Therefore, the simulated electricity price peaks in years 2017 and 2018 coincide with the changed estimated merit order profile of the gas and lignite generation. The estimated merit order profiles in these years become much more explicit and with notable slope changes near the full capacity utilisation (Fig. 3). The simulation results confirm reduced price volatility with excluded RES generation. With the simulation results, we can confirm that the RES generation has much higher impact on the electricity price reduction if the electricity price setting occurs at the steep profile of the merit order. The highest electricity price increase between 2.15 and 2.72 €/MWh is simulated in year 2017. The highest electricity price increase coincides with the lowest reported hydro generation in Romania and structural change in the German electricity price. The German electricity price was on average 5 €/MWh higher in year 2017 compared to the year 2016 (Table 1). In year 2018, the German electricity price rose an additional 7 €/MWh. The change in the estimated Romanian gas and lignite supply curve profiles coincides with a rise of German electricity prices. Therefore, we conclude that the rise in Romanian electricity prices is associated with higher short-term marginal costs of electricity generation. Further analysis is beyond the scope of this paper.

The simulated electricity price and standard deviation are on average reduced in all three countries. Due to the lower RES generation share in the Hungarian generation mix (Fig. 2), simulation results indicate minor price changes and standard deviation reductions. Therefore, simulation results are more representative in the Greek and Romanian electricity markets, as the price increments are in the range 0.2–3.26 €/MWh, and with notably reduced standard deviations. The overall simulation robustness is provided, as the general conclusions do not depend on the selected merit order forecasting algorithm.

## Discussion

Würzburg et al. (2013) classify MOE studies in the simulation-based and empirical analysis studies. In our study, the initial empirical confirmation and ceteris paribus quantification of the MOE is performed by the frequently practised econometric approach. The adjustment of the realised day-ahead prices to the no-RES generation scenario is simulated according to the estimated power plant merit order. Simulation-based studies typically rely on solving information rich and flexible simulation models used for power system or agent-based simulations (Troha and Hauser 2015; Schill et al. 2017). Due to the limited public data availability in the analysed country scope discussed in the previous sections, assembly of such simulation-based studies was not feasible. Therefore, modern statistical methods are used to bridge this gap in the preparation of the no-RES generation simulation. A family of data mining algorithms is used to estimate the power plant merit order. The estimated energy imbalance caused by the excluded RES generation is compensated by the additional conventional generation dispatch according to the estimated power plant merit order that sets a new electricity price.

Due to the fundamental difference in electricity generation mixes, interconnection properties, and approaches to the analysis, the comparison of obtained results from different studies could be misleading and should be done with special care. Therefore, we limit discussion section to the MOE econometric confirmation, as the ceteris paribus quantification of the MOE is a characteristic of the individual electricity market. In the existing literature focused on the key EU energy markets, MOE is econometrically confirmed by the negative sign and statistical significance of the explanatory variable indicating the effect of RES generation on electricity prices. Based on the estimated econometric models, we confirmed MOE effect in Hungarian, Romanian, and Greek electricity markets. In Greece, we could not find significant difference between the coefficients for solar and wind generation, and therefore the price effects seem to be very similar. Conversely, the Romanian solar generation coefficient turned out to be positive. The positive correlation between the pronounced summer solar generation peak and high electricity prices might have influenced model estimation. Wind is the only reported RES source in the Hungarian electricity system; therefore, the distinction between the effect of solar and wind generation on electricity prices is not applicable.

The estimated MOE effect in Hungary and Greece is higher on the high-load days compared to the low-load days. A similar effect is reported by Würzburg et al. (2013), Sensfuß et al. (2008), and Weigt (2009). In contrast, the estimated MOE effect in Romanian electricity market is higher on low-load days. One possible explanation for this contradicting phenomenon is a steeper profile of the lignite supply curve. The estimated lignite supply curve has a steeper profile already in the lower-quantity area, compared to the estimated Hungarian and Greek lignite supply curves (Fig. 3). The electricity price setting on low-load days occurs in the lower-quantity area; therefore, a pronounced MOE effect could be justified by the steep merit order profile in the price setting area. The econometric findings are in accord to the estimated merit order profiles. Therefore, estimation of the power plant merit order by the modern statistical methods turned out to provide invaluable reasoning insights to the econometrically estimated results.

The simulation results of the no-RES generation scenario on average suggest insignificant changes of the Hungarian realised day-ahead price adjusted to the no-RES generation scenario. The Greek and Romanian electricity markets, with higher RES generation shares in their electricity generation mix, empirically qualify as more interesting case studies. In line with the economic theory, the simulation results indicate significant price increments in the no-RES generation scenario in both countries. Additionally, reduced price volatility is found due to eliminated intermittent RES generation.

Simulation robustness of our no-RES generation simulation is proved as the general conclusions do not depend on the selected merit order forecasting algorithm. On average, we confirm higher electricity prices and lower price volatility. Further, impact of RES generation is more profound with higher electricity prices, i.e. higher short-term marginal costs of production. Supply side dynamics associated with profit optimisation is due to the limited public data availability approximated with modern statistical methods and requires special attention in future research. The simulation approach, with the direct control of the short-term electricity production marginal costs, would provide additional valuable insights into the merit order data generation process.

## Conclusion

With the empirical analysis and no-RES generation simulation, we confirm economic theory predictions that an increase in RES generation in the short run reduces the electricity price in the Hungarian, Greek, and Romanian electricity markets. National promotion strategies triggered by the Directive 2001/77/EC on renewable energies in the electricity sector are considered as the main reason for this development. All EU member states have introduced policies to support the market introduction of RES generation. Therefore, our research paper supplements and verifies existing literature findings focused on the investigation of the effects of installed renewable capacity on electricity market development.

Econometric models confirmed statistically significant MOE in all analysed countries. The RES generation effect on the electricity price levels primarily depends on the individual power system characteristics. Econometrically estimated MOE is quantitatively interpreted as a price reduction in €/MWh for each additional GWh of renewable generation. Therefore, the estimated merit order effect is much larger in the smaller Hungarian power system, compared to the bigger Greek and Romanian power systems. The estimated MOE effect is stable throughout different model variations and in line with the reviewed literature findings. In the Romanian electricity market, we found an exception, as the solar generation turned out to be positively correlated with electricity prices. The positive correlation between the pronounced summer solar generation peak and high electricity prices might have influenced model estimation with differentiated RES sources. In the Hungarian and Greek electricity markets, we found a pronounced MOE on high-load days, whereas in Romania the effect is more pronounced on low-load days. The estimated supply curves for each generation technology provided valuable insights to assist the reasoning behind the estimated econometric coefficients.

Simulation of the no-RES generation scenario accounts for the international flow dynamics and adaptation of the conventional generation dispatch to the omitted RES generation. The estimated energy imbalance in the no-RES generation scenario, caused by the excluded RES generation, is compensated by the additional conventional generation dispatch according to the estimated power plant merit order. A family of data mining algorithms applied for the merit order estimation suitably handled non-linear behaviour of the electricity price signals and bridged gap in limited data availability. The impact of RES generation on country net export is estimated by the multivariate regression model and empirically reveals that RES generation stimulates foreign demand. We confirmed price increments due to the excluded RES generation in all three countries. In addition, the reduced standard deviation in the no-RES generation scenario is a result of omitted volatile RES generation. Simulation robustness is proved as the general conclusions do not depend on the selected merit order estimation algorithm. Econometric MOE confirmation and supporting simulation framework turned out to be successful combination, as the estimated power system merit order profiles support results from econometric models.

Based on the findings presented in the article, policymakers in Central and South East European countries, where the electricity markets are deemed less mature, should prioritise implementing policies that promote the integration of renewable energy sources into their electricity markets. The established downward pressure on wholesale electricity prices is less pronounced in countries with low penetration of renewables and high interconnection capacities. While lower electricity prices are beneficial for final consumers, supporting renewables can also cause market distortions and have adverse impact on economic viability of conventional power plants. Designed policy measures therefore need to consider the existing production mix and interconnection capacities, combined with simulation results to ascertain the optimal shares of production technologies in the generation mix. In doing so, it is necessary to account for the development dynamics in regionally interconnected electricity systems, as well as at the European level. Therefore, a more holistic approach is recommended by taking into account country specifics. For example, our results suggest that there are already too many solar power plants in Romania and it would make more sense to support other technologies, while on the other hand, there is still room for solar power plants in Greece. In the light of increasingly growing shares of renewables in the EU, there is currently an ongoing debate on improving efficiency of renewable investment support schemes and limiting their use to the needs assessed. While there is admittedly no one-size-fits-all approach to such support frameworks, the most valuable projects would not necessarily be those that produce more electricity in total, but those that produce more, where and when it is most valuable for the system (ACER 2022). Additionally, given data constraints, investing in data collection and sharing initiatives, coupled with the application of modern statistical methods, will enhance the accuracy of simulation models and inform policy decisions.

## Data Availability

Datasets related to this article can be found at https://transparency.entsoe.eu, hosted by the ENTSO-E Transparency Platform.

## Appendix

Tables 4, 5, 6, 7, 8, 9, 10, 11, 12, 13, 14, 15, and 16

**Table 4:** Observed maximum generation mix penetrations in MW

Country	Type	2015	2016	2017	2018-Q3
GR	Gas	3733	3492	3850	4310
GR	Hydro	1979	2080	1961	2260
GR	Lignite	4110	3808	5150	3651
GR	Other	2	2	0	0
GR	Solar	2062	1923	1847	1933
GR	Wind	1412	1330	1702	1695
HU	Gas	854.25	800.75	770.5	763
HU	Hydro	26.5	27	26	26.25
HU	Lignite	1502.25	1651.25	1948.75	1681.50
HU	Nuclear	1941.00	1937.75	1939.00	1939.00
HU	Other	458.25	464.75	432.75	164
HU	Wind	304.75	306	301	298.25
RO	Gas	3466	3635	3523	3007
RO	Hydro	4330	4691	3985	4416
RO	Lignite	2283	2390	2441	2147
RO	Nuclear	1426	1420	1433	1415
RO	Other	78	74	70	64
RO	Solar	774	807	847	864
RO	Wind	2686	2795	2756	2750

Source: ENTSO-E TP (2020)

**Table 5:** OLS estimation of daily changes in Greek electricity prices

	Model 1	Model 2	Model 3	Model 4	Model 5	Model 6	Model 7	Model 8
	Year 2015	Year 2016	Year 2017	Year 2018	All years	All years Ren split	All years High load	All years Low load
	*∆Pelec, t*	*∆Pelec, t*	*∆Pelec, t*	*∆Pelec, t*	*∆Pelec, t*	*∆Pelec, t*	*∆Pelec, t*	*∆Pelec, t*
*∆Pelec, d* − 1	0.382 [0.00***]	0.390 [0.00***]	0.645 [0.00***]	0.672 [0.00***]	0.704 [0.00***]	0.707 [0.00***]	0.677 [0.00***]	0.578 [0.00***]
*∆DE, d*	0.065 [0.01***]	0.107 [0.00***]	0.199 [0.00***]	0.156 [0.00***]	0.160 [0.00***]	0.161 [0.00***]	0.226 [0.00***]	0.107 [0.00***]
*∆Load, d*	0.003 [0.00***]	0.002 [0.00***]	0.002 [0.00***]	0.001 [0.07]	0.001 [0.00***]	0.001 [0.00***]	0.002 [0.00***]	0.003 [0.00***]
*∆Ren, d*	− 0.005 [0.00***]	− 0.005 [0.00***]	− 0.006 [0.00***]	− 0.005 [0.00***]	− 0.004 [0.00***]	/	− 0.005 [0.00***]	− 0.003 [0.00***]
*∆Wind, d*	/	/	/	/	/	− 0.004 [0.00***]	/	/
*∆Solar, d*	/	/	/	/	/	− 0.003 [0.00***]	/	/
*R*^2^	0.59	0.50	0.76	0.78	0.77	0.77	0.80	0.57
Adjusted *R*^2^	0.59	0.49	0.76	0.78	0.77	0.77	0.80	0.57
*F*-test	130.80	88.34	291.30	233.72	1127.29	903.12	330.65	112.90
*p* value (*F*)	0.00***	0.00***	0.00***	0.00***	0.00***	0.00***	0.00***	0.00***

“***” indicating significance at 1% level, and *p* values in [] brackets

**Table 6:** OLS estimation of daily changes in Hungarian electricity prices

	Model 1	Model 2	Model 3	Model 4	Model 5	Model 6	Model 7	Model 8
	Year 2015	Year 2016	Year 2017	Year 2018	All years	All years Ren split	All years High load	All years Low load
	*∆Pelec, t*	*∆Pelec, t*	*∆Pelec, t*	*∆Pelec, t*	*∆Pelec, t*	*∆Pelec, t*	*∆Pelec, t*	*∆Pelec, t*
*∆Pelec, d* − 1	0.469 [0.00***]	0.549 [0.00***]	0.501 [0.00***]	0.424 [0.00***]	0.582 [0.00***]	0.582 [0.00***]	0.689 [0.00***]	0.563 [0.00***]
*∆DE, d*	0.207 [0.00***]	0.305 [0.00***]	0.228 [0.00***]	0.531 [0.00***]	0.313 [0.00***]	0.313 [0.00***]	0.258 [0.00***]	0.262 [0.00***]
*∆Load, d*	0.008 [0.00***]	0.004 [0.00***]	0.013 [0.00***]	0.003 [0.00***]	0.006 [0.00***]	0.006 [0.00***]	0.008 [0.00***]	0.005 [0.00***]
*∆Ren, d*	− 0.015 [0.00***]	− 0.009 [0.05]	− 0.035 [0.00***]	− 0.005 [0.47]	− 0.013 [0.00***]	/	− 0.014 [0.07]	− 0.009 [0.08]
*∆Wind, d*	/	/	/	/	/	− 0.013 [0.00***]	/	/
*∆Solar, d*	/	/	/	/	/	/	/	/
*R*^2^	0.63	0.71	0.74	0.82	0.75	0.75	0.74	0.70
Adjusted *R*^2^	0.63	0.71	0.73	0.81	0.75	0.75	0.74	0.70
*F*-test	152.8	218.78	248.39	294.11	994.96	994.96	235.97	194.62
*p* value (*F*)	0.00***	0.00***	0.00***	0.00***	0.00***	0.00***	0.00***	0.00***

“***” indicating significance at 1% level, and *p* values in [] brackets

**Table 7:** OLS estimation of daily changes in Romanian electricity prices

	Model 1	Model 2	Model 3	Model 4	Model 5	Model 6	Model 7	Model 8
	Year 2015	Year 2016	Year 2017	Year 2018	All years	All years Ren split	All years High load	All years Low load
	*∆Pelec, t*	*∆Pelec, t*	*∆Pelec, t*	*∆Pelec, t*	*∆Pelec, t*	*∆Pelec, t*	*∆Pelec, t*	*∆Pelec, t*
*∆Pelec, d* − 1	0.362 [0.00***]	0.414 [0.00***]	0.433 [0.00***]	0.497 [0.00***]	0.549 [0.00***]	0.545 [0.00***]	0.633 [0.00***]	0.452 [0.00***]
*∆DE, d*	0.243 [0.00***]	0.209 [0.00***]	0.294 [0.00***]	0.386 [0.00***]	0.283 [0.00***]	0.254 [0.00***]	0.251 [0.00***]	0.262 [0.00***]
*∆Load, d*	0.004 [0.00***]	0.005 [0.00***]	0.008 [0.00***]	0.003 [0.00***]	0.004 [0.00***]	0.006 [0.00***]	0.005 [0.00***]	0.007 [0.00***]
*∆Ren, d*	− 0.008 [0.00***]	− 0.006 [0.00***]	− 0.011 [0.00***]	− 0.008 [0.00***]	− 0.007 [0.00***]	/	− 0.007 [0.00***]	− 0.008 [0.00***]
*∆Wind, d*	/	/	/	/	/	− 0.007 [0.00***]	/	/
*∆Solar, d*	/	/	/	/	/	0.015 [0.00***]	/	/
*R*^2^	0.68	0.68	0.73	0.73	0.72	0.73	0.72	0.71
Adjusted *R*^2^	0.67	0.67	0.72	0.73	0.72	0.73	0.72	0.7
*F*-test	186.68	185.74	226.84	178.19	846.03	708.57	210.61	197.77
*p* value (*F*)	0.00***	0.00***	0.00***	0.00***	0.00***	0.00***	0.00***	0.00***

“***” indicating significance at 1% level, and *p* values in [] brackets

**Table 8:** Testing for residual heteroscedasticity

Studentised Breusch-Pagan test	GR	HU	RO
Test statistic	112.80	78.97	58.30
df	4	4	4
*p* value	0.00	0.00	0.00

**Table 9:** Testing for residual normality

Test	GR	HU	RO
Shapiro–Wilk	0.906 [0.00***]	0.904 [0.00***]	0.963 [0.00***]
Kolmogorov–Smirnov	0.090 [0.00***]	0.088 [0.00***]	0.063 [0.00***]
Cramer-von Mises	103.166 [0.00***]	113.896 [0.00***]	101.271 [0.00***]
Anderson–Darling	24.697 [0.00***]	22.763 [0.00***]	10.022 [0.00***]

^***^ indicating significance at 1% level, and *p* values in [] brackets

**Table 10:** Testing for multicollinearity

VIF	GR	HU	RO
*∆Pelec, t* − 1	1.372	1.336	1.254
*∆DE, t*	1.296	1.612	1.365
*∆Load, t*	1.201	1.422	1.334
*∆Ren, t*	1.024	1.053	1.089

**Table 11:** Testing for residual autocorrelation

Durbin-Watson test	GR	HU	RO
Test statistic	2.08	1.74	1.66
*p* value	0.92	0.00	0.00

**Table 12:** OLS estimation of daily changes in electricity prices (robust standard errors)

Model	GR	HU	RO
	*∆Pelec, t*	*∆Pelec, t*	*∆Pelec, t*
*∆Pelec, t* − 1	0.704 [0.00***]	0.582 [0.00***]	0.549 [0.00***]
*∆DE, t*	0.160 [0.00***]	0.313 [0.00***]	0.283 [0.00***]
*∆Load, t*	0.001 [0.00***]	0.006 [0.00***]	0.004 [0.00***]
*∆Ren, t*	− 0.004 [0.00***]	− 0.013 [0.00***]	− 0.007 [0.00***]
*R*^2^	0.77	0.75	0.72
Adjusted *R*^2^	0.77	0.75	0.72
*F*-test	335.80	641.70	502.5
*p* value (*F*)	0.00	0.00	0.00

^***^ indicating significance at 1% level, and *p* values in [] brackets

**Table 13:** OLS estimation of daily changes in electricity prices (additional lag of dependent variable)

Model	GR	HU	RO
	*∆Pelec, t*	*∆Pelec, t*	*∆Pelec, t*
*∆Pelec, t* − 1	0.541 [0.00***]	0.483 [0.00***]	0.428 [0.00***]
*∆Pelec, t* − 2	0.192 [0.00***]	0.121 [0.00***]	0.160 [0.00***]
*∆DE, t*	0.163 [0.00***]	0.310 [0.00***]	0.279 [0.00***]
*∆Load, t*	0.001 [0.00***]	0.007 [0.00***]	0.004 [0.00***]
*∆Ren, t*	− 0.005 [0.00***]	− 0.013 [0.00***]	− 0.008 [0.00***]
*R*^2^	0.78	0.75	0.73
Adjusted *R*^2^	0.78	0.75	0.73
*F*-test	967.50	822.30	718.40
*p* value (*F*)	0.00	0.00	0.00

^***^ indicating significance at 1% level, and *p* values in [] brackets

**Table 14:** OLS estimation of net export in Hungary

	Year 2015	Year 2016	Year 2017	Year 2018
	*NX, t*	*NX, t*	*NX, t*	*NX, t*
*NX, t* − 24	0.860 [0.00***]	0.860 [0.00***]	0.847 [0.00***]	0.886 [0.00***]
*RES, t*	0.427 [0.00***]	0.626 [0.00***]	0.849 [0.00***]	0.695 [0.00***]
*R*^2^	0.75	0.75	0.74	0.78
Adjusted *R*^2^	0.75	0.75	0.74	0.78
*F*-test	13,091.44	12,891.61	12,407.85	12,122.70
*p* value (*F*)	0.00	0.00	0.00	0.00

“***” indicating significance at 1% level, and *p* values in [] brackets

**Table 15:** OLS estimation of net export in Greece

	Year 2015	Year 2016	Year 2017	Year 2018
	*NX, t*	*NX, t*	*NX, t*	*NX, t*
*NX, t* − 24	0.750 [0.00***]	0.811 [0.00***]	0.796 [0.00***]	0.759 [0.00***]
*RES, t*	0.048 [0.00***]	0.052 [0.00***]	0.055 [0.00***]	0.045 [0.00***]
*R*^2^	0.59	0.66	0.66	0.59
Adjusted *R*^2^	0.59	0.66	0.66	0.59
*F*-test	2814.29	7426.11	8053.16	4659.02
*p* value (*F*)	0.00	0.00	0.00	0.00

“***” indicating significance at 1% level, and *p* values in [] brackets

**Table 16:** OLS estimation of net export in Romania

	Year 2015	Year 2016	Year 2017	Year 2018
	*NX, t*	*NX, t*	*NX, t*	*NX, t*
*NX, t* − 24	0.458 [0.00***]	0.528 [0.00***]	0.363 [0.00***]	0.446 [0.00***]
*RES, t*	0.330 [0.00***]	0.424 [0.00***]	0.513 [0.00***]	0.393 [0.00***]
*R*^2^	0.55	0.58	0.57	0.48
Adjusted *R*^2^	0.55	0.58	0.57	0.48
*F*-test	5208.98	6010.65	5664.70	3118.52
*p* value (*F*)	0.00	0.00	0.00	0.00

“***” indicating significance at 1% level, and *p* values in [] brackets

Figures 4 and 5

## Footnotes

1 Troha and Hauser (in Schill and Gerbaulet 2015) used unit commitment model to evaluate the impact of start-up costs and grid operator on the UK power price equilibrium. Schill et al. investigated the impact of fluctuating RES generation on the start-up costs in Germany (Schill et al. 2017).

2 Unit commitment models are reliable and efficient, but require exact set of data for each individual power plant (generation data, outages data, start-up cost, efficiency factor, fuel prices, CO_2_ price, etc.).

3 For a detailed description of the KNN algorithm, please refer to Mangalova and Agafonov (2014).

4 For a detailed description of the M5P algorithm, please refer to Wang and Witten (1996).

5 For a detailed description of the RFR algorithm, please refer to Breiman (2001).

6 For general review of generation costs for different electricity generation technologies, please refer to Schröder et al. (2013).

7 A free parameter applicable to this specific model application, “*k*” (number of nearest neighbours), is set to 20. For detailed algorithm description, please refer to Mangalova and Agafonov (2014).

8 A free parameter applicable to this specific model application, “number of trees”, is set to 20. For a detailed RFR algorithm description, please refer to Breiman (2001).

## References

[CR1] ACER (2022) ACER’s final assessment of the EU wholesale electricity market design. April 2022. European Union Agency for the Cooperation of Energy Regulators

[CR2] Azofra D, Jiménez E, Martínez E (2014). Wind power merit-order and feed-in-tariffs effect: a variability analysis of the Spanish electricity market. Energy Convers Manag.

[CR3] Benhmad F, Percebois J (2018). An econometric analysis of the merit-order effect in electricity spot price: the Germany case. Springer International Publishing.

[CR4] Breiman L (2001). Random forests. Mach Learn.

[CR5] Bunn DW, Gianfreda A (2010). Integration and shock transmissions across European electricity forward markets. Energy Econ.

[CR6] Cerjan M, Krzelj I, Vidak M, Delimar M (2013). A literature review with statistical analysis of electricity price forecasting methods. EUROCON.

[CR7] Clò S, Cataldi A, Zoppoli P (2015). The merit-order effect in the Italian power market: the impact of solar and wind generation on national wholesale electricity prices. Energy Policy.

[CR8] Cludius J, Hermann H, Matthes FC, Graichen V (2014). The merit order effect of wind and photovoltaic electricity generation in Germany 2008–2016 estimation and distributional implications. Energy Econ.

[CR9] Croonenbroeck C, Palm M (2020). A spatio-temporal Durbin fixed effects IV-model for ENTSO-E electricity flows analysis. Renew Energy.

[CR10] Deane P, Collins S, Gallachóir BÓ, Eid C, Hartel R, Keles D, Fichtner W (2017) Impact on electricity markets: merit order effect of renewable energies. Europe’s energy transition, Academic Press, pp. 105–118. 10.1016/B978-0-12-809806-6.00016-X

[CR11] Dong S, Li H, Wallin F (2019). Volatility of electricity price in Denmark and Sweden. Energy Procedia.

[CR12] ENTSO-E TP (2020) ENTSO-E transparency platform knowledge base. In: Entso-E. https://transparency.entsoe.eu/. Accessed 1 Dec 2020

[CR13] Figueiredo NC, da Silva PP (2019). The “merit-order effect” of wind and solar power: volatility and determinants. Renew Sustain Energy Rev.

[CR14] Fleschutz M, Bohlayer M, Braun M, Henze G, Murphy MD (2021). The effect of price-based demand response on carbon emissions in European electricity markets: the importance of adequate carbon prices. Appl Energy.

[CR15] Fürsch M, Malischek R, Lindenberger D (2012) Der Merit-Order-Effekt der erneuerbaren Energien - Analyse der kurzen und langen Frist. Institute of Energy Economics at the University of Cologne (EWI), Köln

[CR16] Gelabert L, Labandeira X, Linares P (2011). An ex-post analysis of the effect of renewables and cogeneration on Spanish electricity prices. Energy Econ.

[CR17] Gil HA, Gomez-Quiles C, Riquelme J (2012). Large-scale wind power integration and wholesale electricity trading benefits: estimation via an ex post approach. Energy Policy.

[CR18] Hirth L, Mühlenpfordt J, Bulkeley M (2018). The ENTSO-E Transparency Platform – a review of Europe’s most ambitious electricity data platform. Appl Energy.

[CR19] Janda K (2018). Slovak electricity market and the price merit order effect of photovoltaics. Energy Policy.

[CR20] Jensen SG, Skytte K (2002). Interactions between the power and green certificate markets. Energy Policy.

[CR21] Jónsson T, Pinson P, Madsen H (2010). On the market impact of wind energy forecasts. Energy Econ.

[CR22] Keles D, Genoese M, Möst D (2013). A combined modeling approach for wind power feed-in and electricity spot prices. Energy Policy.

[CR23] Lindström E, Regland F (2012). Modeling extreme dependence between European electricity markets. Energy Econ.

[CR24] Loumakis S, Giannini E, Maroulis Z (2019). Merit order effect modeling: the case of the Hellenic electricity market. Energies.

[CR25] Luňáčková P, Průša J, Janda K (2017). The merit order effect of Czech photovoltaic plants. Energy Policy.

[CR26] Macedo DP, Marques AC, Damette O (2020) The impact of the integration of renewable energy sources in the electricity price formation: is the merit-order effect occurring in Portugal?. Util Policy 66. 10.1016/j.jup.2020.101080

[CR27] Macedo DP, Marques AC, Damette O (2021) The merit-order effect on the Swedish bidding zone with the highest electricity flow in the Elspot market. Energy Econ 102. 10.1016/j.eneco.2021.105465

[CR28] Mangalova E, Agafonov E (2014). Wind power forecasting using the k-nearest neighbors algorithm. Int J Forecast.

[CR29] McConnell D, Hearps P, Eales D, Sandiford M, Dunn R, Wright M, Bateman L (2013). Retrospective modeling of the merit-order effect on wholesale electricity prices from distributed photovoltaic generation in the Australian National Electricity Market. Energy Policy.

[CR30] Neubarth J, Woll O, Weber C, Gerecht M (2006). Beeinflussung der Spotmarktpreise durch Windstromerzeugung. Energiewirtschaftliche Tagesfragen.

[CR31] Pradhan AK, Rout S, Khan IA (2021). Does market concentration affect wholesale electricity prices? An analysis of the Indian electricity sector in the COVID-19 pandemic context. Util Policy.

[CR32] Ragwitz M, Held A (2007) OPTRES. Assessment and optimisation of renewable energy support schemes in the European electricity market. Fraunhofer ISI, Karlsruhe

[CR33] Sachan A, Sahu UK, Pradhan AK, Thomas R (2023). Examining the drivers of renewable energy consumption: evidence from BRICS nations. Renew Energy.

[CR34] Sáenz de Miera G, del Río GP, Vizcaíno I (2008). Analysing the impact of renewable electricity support schemes on power prices: the case of wind electricity in Spain. Energy Policy.

[CR35] Schill WP, Gerbaulet C (2015). Power system impacts of electric vehicles in Germany: charging with coal or renewables?. Appl Energy.

[CR36] Schill WP, Pahle M, Gambardella C (2017). Start-up costs of thermal power plants in markets with increasing shares of variable renewable generation. Nat Energy.

[CR37] Schröder A, Kunz F, Meiss J (2013). Current and prospective costs of electricity generation until 2050.

[CR38] Sensfuß F, Ragwitz M, Genoese M (2008). The merit-order effect: a detailed analysis of the price effect of renewable electricity generation on spot market prices in Germany. Energy Policy.

[CR39] Traber T, Kemfert C (2009). Impacts of the German support for renewable energy on electricity prices, emissions, and firms. Energy J.

[CR40] Transelectrica (2020) Transelectrica - required transparency of generation. https://www.transelectrica.ro/en/web/tel/productie. Accessed 4 Aug 2021

[CR41] Troha M, Hauser R (2015). Calculation of a term structure power price equilibrium with ramping constraints. J Energy Mark.

[CR42] Unger EA, Ulfarsson GF, Gardarsson SM, Matthiasson T (2018). The effect of wind energy production on cross-border electricity pricing: the case of western Denmark in the Nord Pool market. Econ Anal Policy.

[CR43] Wang Y, Witten IH (1996). Introduction of model trees for predicting continuous classes.

[CR44] Weigt H (2009). Germany’s wind energy: the potential for fossil capacity replacement and cost saving. Appl Energy.

[CR45] Weron R (2014). Electricity price forecasting: a review of the state-of-the-art with a look into the future. Int J Forecast.

[CR46] Woll O, Weber C (2012) Merit-Order-Effekte Von Erneuerbaren Energien – Zu Schön Um Wahr Zu Sein? SSRN Electronic Journal 1–11. 10.2139/ssrn.1656926

[CR47] Würzburg K, Labandeira X, Linares P (2013). Renewable generation and electricity prices: taking stock and new evidence for Germany and Austria. Energy Econ.

[CR48] Ziel F, Steinert R, Husmann S (2015). Forecasting day ahead electricity spot prices: the impact of the EXAA to other European electricity markets. Energy Econ.

